# Investigating Death by PowerPoint: Do Medical School Lecturers Adhere to the Cognitive Theory of Multimedia Learning in Their Slide Design?

**DOI:** 10.1111/tct.70315

**Published:** 2025-12-10

**Authors:** Rajin Le Blanc, Nicola Cooper

**Affiliations:** ^1^ Education Centre, School of Medicine University of Nottingham Nottingham UK

**Keywords:** cognitive theory of multimedia learning, lecture, slide design

## Abstract

**Background:**

Lecturers in higher education commonly use slide software like Microsoft PowerPoint. Mayer's cognitive theory of multimedia learning (CTML) describes 15 principles for helping people learn better with words and images and is supported by a large number of empirical studies. Medical school curricula are intensive so teaching should be as effective as possible. Though there is existing research into lectures, this does not specifically determine whether CTML principles are being adopted. This study investigated to what extent lecturers incorporated the principles of CTML into lecture slide design at a single UK medical school.

**Methods:**

Lectures were observed both live and recorded. Based on CTML principles, this included the time students were exposed to text‐heavy (> 10 words) versus text‐light (≤ 10 words) slides; whether images were used; the use of outlines, highlighting and pointing; extraneous images; and the labelling and timing of images. Word counts for slide sets were also recorded.

**Results:**

Students were exposed to text‐heavy slides 84.4% of the time. Forty percent of lectures used outlines at the beginning. Slide sets contained a median of 1531 words and a mean of 38.3 words per slide.

**Conclusion:**

Slide design appeared to consistently violate CTML principles; therefore, lecturers should receive training in adhering to these principles. Future research should examine what barriers exist to adopting CTML principles and how such training for teachers on these principles could be delivered.

## 1 Introduction

The lecture is one of the oldest modalities in teaching. Lectures in higher education commonly use slide software like Microsoft PowerPoint [1]. However, ‘death by PowerPoint’—the use of bullet points and large amounts of text on slides—has been highlighted as an issue within education, academia and business for at least two decades [2, 3, 4, 5].

Multimedia learning is defined as learning with words and images. In the 1980s, Richard E. Mayer's cognitive theory of multimedia learning (CTML) described 15 principles for helping people learn better with words and images [6]. CTML is grounded in cognitive load theory [7] and the dual‐channel principle [8]. Cognitive load theory itself is based on the limited capacity principle [9], which states that humans have a limited capacity to process information; cognitive load theory describes these limitations.

Cognitive load theory splits memory into two components—working memory and long‐term memory. Working memory is the information we briefly hold in our minds at the time we are manipulating it. It is limited to less than 30 s and to 7 ± 2 ideas. Cognitive load is anything that uses up ‘space’ in working memory. Ideas not transferred from working memory into long‐term memory are lost. The dual‐channel principle [9] states that words and images are processed separately in working memory, via an auditory channel and a visual channel, so using words and *related* images can improve processing and retention, thus reducing cognitive load. Several studies have been conducted that support Mayer's CTML summarised in his book, *Multimedia Learning* [6].

CTML provides evidence‐based principles, which can be applied to slide design in lectures. These principles appear to improve both learner experience [10, 11, 12] and knowledge retention [8, 13, 14]. By contrast, other literature on slide design appears to lack an evidence base [15, 16, 17, 18]. Beyond advice around font type, font size and colour contrast, little reference is made to CTML. Practices such as bullet points, one idea per slide or one slide per minute appear to represent opinion rather than primary research. Using sufficient text to warrant bullet points likely runs contrary to CTML.

A comprehensive review (which would also need non‐academic sources) is beyond the scope of this article, but the literature appears to contain conflicting, mutually exclusive approaches to slide design. As such, even where lecturers adopt practices from the literature, these practices may be based on opinion and be in conflict with primary research. Both within medical education and education generally, what does not appear to have been investigated is whether lecturers in a real‐life context incorporate CTML principles into their slide design.

However, Gonzalez‐Mujico and Lasagabaster examined knowledge‐building practices using multimodal PowerPoint slides in their real‐life context [19]. Despite superficial similarities in terminology (‘multimodal’ vs. ‘multimedia’), this study is not based on CTML. Gonzalez‐Mujico and Lasagabaster's mixed‐methods study used legitimation code theory to examine ‘semantic waves’: where abstract concepts are explained in simpler terms (‘unpacked’) then linked back to the abstract concepts (‘repacked’). This contrasts with the strictly quantitative approach of studies into CTML.

Further, Gonzalez‐Mujico and Lasagabaster do not differentiate between slides with ‘a few words’ and slides with no words. CTML's revised redundancy principle states that speech, images and a few words of text are the most effective combination of images and words. Adding more than a few written words is considered to ‘impair learning’, something outside the scope of Gonzalez‐Mujico and Lasagabaster's study. Additionally, Gonzalez‐Mujico and Lasagabaster did not focus on novices whereas CTML is considered less effective in learners with greater expertise [20].

Thus, though such research in its real‐life context has been conducted, research specifically examining the incorporation of CTML into slide design for lectures delivered to novices in its real‐life context was not found. As such, we wished to explore a novice population who commonly receive lectures and are regularly exposed to slides. We therefore focused on early years medical students. The research question was, ‘To what extent do lecturers who deliver lectures to early years medical students incorporate the principles of the cognitive theory of multimedia learning into lecture slide design?’.

## 2 Materials and Methods

Ethical approval was obtained from the University of Nottingham, UK. The ethical approval was accepted by the ethics committee at the UK university where the study was conducted. For feasibility, only first‐year lectures were observed. Observed lectures were limited to those on material within the medical curriculum. Non‐clinical material was included such as statistics or ethics. Lectures on advisory material (‘Avoiding plagiarism’) or administrative issues (‘Introduction to Year 1’) were excluded. Heavily interactive lectures (e.g., lectures described as flipped classrooms) were excluded.

All lectures were delivered in person. Lectures were either observed live or a recording was reviewed. Access to these lectures was organised by medical school staff; at their request, permission from the lecturer was obtained for attending live lectures but was not required for reviewing recorded lectures. Recordings showed slides and audio, but there was no video of the lecturers themselves. Recorded lectures were assessed in chronological order from the start of the academic year. Lectures observed live were attended based on when the author collecting data (R.L.B.) was available. Only the first lecture of each lecturer was analysed.

Times were recorded in seconds with each lecture allocated a 3000‐s (50‐min) timeslot. If a lecture adhered to CTML principles for the majority of the time that the lecture was being delivered, the lecture was excluded from further analysis and the total number of these excluded lectures was recorded. For each set of slides, word counts, slide number and number of words per slide were also recorded using Microsoft PowerPoint's word count feature.

### 2.1 Relevant CTML Principles

Of the 15 principles of CTML, seven are relevant to slide design and were used to judge the extent to which CTML principles were followed during lectures. These are the following:

- Multimedia: images and words are better than words alone.
- Modality: images and spoken words are better than images and text.
- Redundancy: images and spoken words are better than images, spoken words and text.
- Coherence: excluding extraneous material is better than including it.
- Signalling: including cues to highlight the organisation of material is better than not including them.
  ○ Classic signalling: use of an outline
  ○ Graphic organisation
  ○ Specific pointing: an instructor points to a specific part of an image rather than at the image in general
- Spatial contiguity: corresponding words should be near rather than far from images.
- Temporal contiguity: corresponding words should be presented alongside images rather than successively.

Some CTML principles have ‘boundary conditions’. A boundary condition of a CTML principle is a situation where initial evidence suggested it applied but later evidence demonstrated it does not. This is important as a ‘boundary condition’ of the redundancy principle is that ‘a few words’ of text are better than no text.

The redundancy principle with this boundary condition, the multimedia principle and the modality principle can be combined to form a single principle, which will be referred to as the ‘minimal text principle’ [21]. This principle and the four other principles are the following:

- Minimal text: images, spoken words and a few words of text are the best combination of images, spoken words and text.
- Coherence: excluding extraneous material is better than including it.
- Signalling: including cues to highlight the organisation of material is better than not including them.
  ○ Classic signalling: use of an outline
  ○ Graphic organisation
  ○ Specific pointing: an instructor points to a specific part of an image rather than at the image in general
- Spatial contiguity: corresponding words should be near rather than far from images.
- Temporal contiguity: corresponding words should be presented alongside images rather than successively.

Below, data collection is described with reference to each relevant CTML principle.

(The ‘minimal text principle’ is not a term used in the wider CTML literature. It is used here only as a convenient way to describe the multimedia, modality and redundancy principles together in the context of slide design.)

### 2.2 The Minimal Text Principle—Data Collection

Slides were divided into text‐heavy and text‐light. Text‐heavy slides contained more than 10 words and text‐light slides contained 10 words or fewer based on studies which explored using ‘a few words’ of text [21, 22]. Slides were also divided into those with images and those with no images. This led to four categories of slide:

- Text‐heavy, no images
- Text‐heavy, with images
- Text‐light, no images
- Text‐light, with images.

For the four categories of lecture slides used, the time spent in each category was recorded in seconds (s). In PowerPoint, changes to what appears on screen can be made using two different mechanisms: transitions (changing slides) or animations (changes to what is on the current slide). Category changes were recorded regardless of the mechanism used.

Recorded lectures were not watched in their entirety. Videos were skipped forward in 10‐s increments until there was a change in slide category. When a change occurred, the video was rewound to when this happened and the time recorded. There was a small risk of missing very short periods spent in a different slide category. However, this would require that the period last < 10 s and for that period to fall between two points at which the recording was being reviewed. Taking this risk decreased the time it took to review each lecture and therefore increased the number of lectures it was possible to review. During lectures observed live, times were recorded in real‐time and entire lectures reviewed as these could not be skipped forward.

Any non‐text item on a slide was considered an image. This included graphs, diagrams and tables as well as pictures. However, text contained within these images (such as legends, labels, or figure descriptions) was included when counting the number of words for categorising a slide as text‐heavy or text‐light. CTML does not differentiate between text contained within images and other text.

### 2.3 The Coherence Principle—Data Collection

The number of extraneous images in each lecture was recorded. Each lecture was placed into a group based on the number of extraneous images: lectures with 0, 1–5, 6–10, 11–15, 16–20, 21–25 or 26–30 images. No lecture had more than 30 images. The number of lectures within each group was recorded.

### 2.4 The Signalling Principle—Data Collection

The presence of classic signalling (use of an outline) was recorded. Although the studies examining graphic organisation typically used red words to highlight an idea, any use of word highlighting was recorded. Any examples of specific pointing whether using a cursor or pointing physically were recorded.

### 2.5 The Spatial and Temporal Contiguity Principles—Data Collection

Whether images were mostly directly labelled (spatial contiguity) or discussed at the time of presentation (temporal contiguity) was also recorded.

## 3 Results

Fifty‐two lectures were reviewed comprising six lectures attended in person and 46 lectures reviewed on video. Two video lectures adhered to CTML principles for the majority of the time and were excluded from further analysis, leaving 50 lectures, 44 of these being video lectures.

Lectures lasted a mean length of 2553.4 s (median = 2665 s) with a minimum of 1128 s and a maximum of 3268 s. The range was 2140 s, and the interquartile range was 646.75 s. Each time slot was 3000 s (50 min).

### 3.1 Data Pertaining to the Minimal Text Principle

**FIGURE 1:**
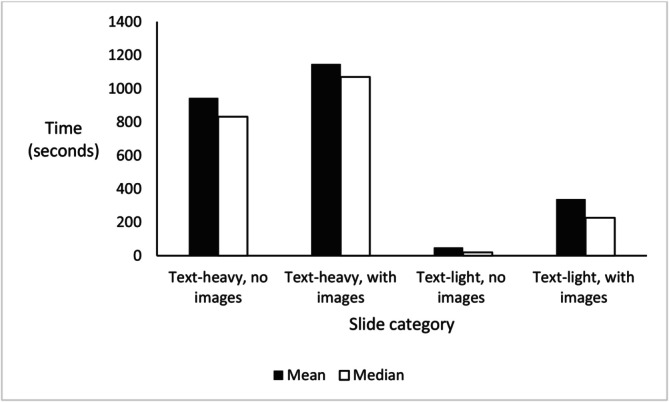
Bar chart showing mean and median times lecturers spent in each slide category in seconds in 50 lectures.

Interactive sections in lectures comprised 3.1% of total lecture time. Excluding interactive time, lecturers spent most time on text‐heavy slides, as shown in Figure 1. Students were exposed to text‐heavy slides 84.4% and slides with images 59.9% of the time. Text‐heavy with images slides were present in all lectures, and the mean number of words per slide was 38.2 (median 38.3).

### 3.2 Data Pertaining to the Coherence Principle

Extraneous images were present in 28/50 (56.0%) lectures. All but one lecture contained between 1 and 5 extraneous images. The remaining lecture was an outlier with 30 images.

### 3.3 Data Pertaining to the Signalling Principle

Classic signalling (an outline) was present in 20/50 (40.0%) lectures. Red words were present in 1/50 (2.0%) lectures, and pointing was present in 1/50 (2.0%) lectures.

### 3.4 Data Pertaining to the Spatial and Temporal Contiguity Principles

Images exhibited spatial contiguity in 49/50 (98.0%) lectures. The remaining lecture's images exhibited spatial contiguity for more than 75% of images. Images exhibited temporal contiguity in 50/50 (100%) lectures.

## 4 Discussion

This study aimed to determine the extent to which lecturers who deliver lectures to early years medical students incorporated the seven CTML principles relevant to slide design. For the purpose of this paper, the ‘minimal text principle’ was described as a practical amalgamation of the multimedia, modality and (with its boundary condition) redundancy principles. This resulted in five principles:

- Minimal text
- Coherence
- Signalling
- Spatial contiguity
- Temporal contiguity

The data from each of the five principles are discussed in turn below followed by a discussion of the implications of this research.

### 4.1 The Minimal Text Principle

Students were exposed to text‐heavy slides 84.4% of the time, an approach that CTML shows impairs learning through violating the minimal text principle [6, 8, 11, 13]. In addition, the mean word count per slide was 38.2—significantly more than the ‘few words’ suggested by this principle. Lecturers did however use images relatively frequently at 59.9% of the time.

In *Multimedia Learning*, Mayer summarises ‘five of five tests’ for the redundancy principle (which underpins the minimal text principle) in ‘system‐controlled’ learning, where learners cannot control the pace of learning, that is, a lecture. This gave a combined median effect size of 0.72 using Cohen's *d* [6]. An effect size of ≥ 0.5 or more is considered medium with ≥ 0.8 considered large [6]. This is without considering the multimedia and modality principles and their effects, which also underpin the minimal text principle.

*Students were exposed to text‐heavy slides 84.4% of the time, an approach which CTML shows impairs learning*.

These effect sizes demonstrate the considerable positive impact adhering to a CTML principle has on learning, let alone adhering to multiple CTML principles, though it is beyond the scope of this study to calculate effect sizes in a similar way. When considered alongside existing studies into CTML and slide design [8, 13, 14], it is likely adhering to the minimal text principle would improve learning for medical students attending these lectures.

### 4.2 The Coherence Principle

Although extraneous images were used in just over half of lectures (56%), this was limited to only one to five images in each lecture (with only one outlier at 30 images). As such, lecturers largely adhered to this principle.

### 4.3 The Signalling Principle

An outline was used in 40% of lectures. This is referred to as ‘classic signalling’ in CTML [6]. First, outlines should be used at the beginning of all lectures. Second, studies into classic signalling go further than outlines alone. They include headings derived from the outline and using verbal pointer words (‘first …’, ‘second …’, ‘third …’) to highlight to learners their place within the outline.

A limitation of this study is that the analysis of headings and pointer words was beyond its scope. Nonetheless, given the evidence is based on the combination of outlines, headings and pointer words, it is reasonable to assume that lecturers should include all 3 of these elements in their lectures.

Only one lecture used the highlighting of words and one other lecture used specific pointing. Another limitation of this study is that only 6 of the 50 lectures were observed live. Although specific pointing was not observed during lectures attended live, it is possible this occurred during recorded lectures where only lecturer audio and slides were available.

### 4.4 The Spatial and Temporal Contiguity Principle

Lecturers in 49/50 lectures almost exclusively used diagrams where items within the diagram were directly labelled rather than using a key or legend, adhering to the spatial contiguity principle. All lecturers explained images as they appeared, adhering to the temporal contiguity principle.

### 4.5 Implications for Lecturers

These results suggest changes lecturers should make to their slide design. The issues were almost exclusively with violating the minimal text principle and the signalling principle. To adhere to the minimal text principle, lecturers should reduce on‐screen text, aiming for only a few words at any given time. Lecturers could also consider increasing their use of images though this may be more appropriate for certain topics (e.g., anatomy, physiology) than others (e.g., ethics).

Suboptimal design may be because slides also serve as notes for the lecturer and student handouts. However, lecturers can use PowerPoint's ‘presenter view’ for their own notes or use paper notes. For example, in the freely available lectures for Harvard's *CS50—Introduction to Computer Science*, Professor David Malan uses paper notes (along with slides mostly containing minimal text) throughout his lectures on the basics of computer programming [23].

This reduces the lecturer's reliance on slides as prompts, allowing the slides to be used solely as visual aids for learners. As they contain similar information, lecturers' notes could be turned into student handouts. Furthermore, lectures are commonly recorded for students to review later (as at the institution where this study was conducted), further reducing the need to use slides as handouts.

Slides can also aid with signalling. To adhere to the signalling principle, first, lecturers should focus on correctly structuring their talks with clear outlines at the beginning, relevant headings taken from those outlines throughout lectures and pointer words to highlight when lecturers are moving from one heading to another. Second, lecturers may wish to consider adding animations to slides that highlight or point to specific items if they do not do so already. This could also be achieved with a laser pointer or the mouse cursor.

### 4.6 Generalisability, Limitations and Strengths

There are important limitations to the generalisability of these results. This was a single‐site study where data were collected by one researcher. Further, this was limited to a single year within one discipline. In addition, medical students frequently come from different educational backgrounds so defining them as novices may be inaccurate.

Nonetheless, as alluded to in the introduction, several articles (both academic and non‐academic) highlight concerns with ‘death by PowerPoint’/text‐heavy slides [2, 3, 4, 5]. These concerns appear longstanding and span education, academia and business but are largely anecdotal. A strength of this study is the collection of primary data within a real‐life context; these concerns appear to have at least some merit.

*Several articles … highlight concerns with ‘death by PowerPoint’/text‐heavy slides … and span education, academia and business*.

Whether this research applies to a particular institution needs to be considered case by case. For instance, a fellow UK medical school with a large number of lectures in its early years is likely to have more similarities to this study than a graduate‐entry arts or humanities course outside the United Kingdom.

CTML principles and their application to lectures slide design are nevertheless not specific to medical education. Any institution where lecture slides are text‐heavy or lectures are ineffectively structured should consider the suggestions above. Further, such institutions should consider how their lecturers are trained in designing slides.

*Institutions should consider how their lecturers are trained in designing slides*.

### 4.7 Avenues for Future Research

Despite a significant body of literature on slide design and death by PowerPoint, articles giving advice or raising concerns are frequently based on opinion [2, 3, 4, 5, 15, 16, 17, 18]. Despite this study's limitations, particularly in terms of scope and size, the underlying approach of examining slides in their real‐life context using an existing, evidence‐based theoretical framework (in this case CTML) could be expanded. Though a different context and theoretical framework, Gonzalez‐Mujico and Lasagabaster's study achieved this, demonstrating that more rigorous research into slide design is warranted. More objective and rigorous slide analysis may be achievable with programs utilising optical character recognition.

Further research is also warranted into how and why lecturers design slides in the way they do. This could include: research into their—if any—current training; current beliefs on slide design; and how slide design is affected by demographics and experience. In addition, other aspects of lecturing such as public speaking could be researched using either CTML or other theoretical frameworks.

## 5 Conclusion

CTML principles have been shown to help people learn better [6] including when applied to lecture slide design [8, 13, 14]. This study found that lecturers who deliver lectures to early years medical students frequently violated these principles. This suggests lecturers should reduce the amount of text on slides and be more rigorous in displaying the structure of their lectures. This has implications for the effectiveness of teaching and learning at medical school, with its intensive curriculum. There are possible implications for any institution where lectures are delivered, but this needs careful consideration of the similarities and differences between such institutions and the institution in this study. Further research into slide design in its real‐life context based on evidence‐based theories is warranted in addition to research into training in, beliefs around and experience of slide design.

## Author Contributions

**Rajin Le Blanc:** conceptualization, investigation, writing – original draft, writing – review and editing, methodology, formal analysis, data curation, validation. **Nicola Cooper:** conceptualization, supervision, writing – review and editing.

## Funding

The authors have nothing to report.

## Ethics Statement

Faculty of Medicine and Health Sciences Research Ethics Committee, University of Nottingham UK, 10 May 2023. Reference Number FMHS 186‐0123. This study was conducted as part of an MMedSci in Medical Education dissertation.

## Conflicts of Interest

The authors declare no conflicts of interest.

## Data Availability

The data that support the findings of this study are available on request from the corresponding author. The data are not publicly available due to privacy or ethical restrictions.
